# Automation Pyramid as Constructor for a Complete Digital Twin, Case Study: A Didactic Manufacturing System

**DOI:** 10.3390/s21144656

**Published:** 2021-07-07

**Authors:** Edwin Mauricio Martinez, Pedro Ponce, Israel Macias, Arturo Molina

**Affiliations:** Tecnológico de Monterrey, Department of Mechatronics, School of Engineering and Sciences, Mexico City Campus, Calle del Puente 222, Tlalpan, México City 14380, Mexico; a01657191@itesm.mx (E.M.M.); israel.macias@tec.mx (I.M.); armolina@tec.mx (A.M.)

**Keywords:** digital twin, automation pyramid, industry 4.0, innovative products, manufacturing model, educational innovation, higher education

## Abstract

Nowadays, the concept of Industry 4.0 aims to improve factories’ competitiveness. Usually, manufacturing production is guided by standards to segment and distribute its processes and implementations. However, industry 4.0 requires innovative proposals for disruptive technologies that engage the entire production process in factories, not just a partial improvement. One of these disruptive technologies is the Digital Twin (DT). This advanced virtual model runs in real-time and can predict, detect, and classify normal and abnormal operating conditions in factory processes. The Automation Pyramid (AP) is a conceptual element that enables the efficient distribution and connection of different actuators in enterprises, from the shop floor to the decision-making levels. When a DT is deployed into a manufacturing system, generally, the DT focuses on the low-level that is named field level, which includes the physical devices such as controllers, sensors, and so on. Thus, the partial automation based on the DT is accomplished, and the information between all manufacturing stages could be decremented. Hence, to achieve a complete improvement of the manufacturing system, all the automation pyramid levels must be included in the DT concept. An artificial intelligent management system could create an interconnection between them that can manage the information. As a result, this paper proposed a complete DT structure covering all automation pyramid stages using Artificial Intelligence (AI) to model each stage of the AP based on the Digital Twin concept. This work proposes a virtual model for each level of the traditional AP and the interactions among them to flow and control information efficiently. Therefore, the proposed model is a valuable tool in improving all levels of an industrial process. In addition, It is presented a case study where the DT concept for modular workstations underpins the development of technologies within the framework of the Automation Pyramid model is implemented into a didactic manufacturing system.

## 1. Introduction

New smart-connected devices facilitate information flow to different levels of decision-makers. Consequently, the traditional factories are migrating their manufacturing processes to more innovative, optimized enterprises. Thus, the smart factory concept emerges as dramatically intensified manufacturing intelligence applications throughout the manufacturing and supply chain enterprise [1]. Smart factories are called to transform into companies with more dynamic economic and supply chain services determined by business and market demand. Some experts addressing the manufacturing changes attend to customer demands, the nature of products, the production economics, and the value chain economics [2].

Within the smart factory are emerging systems that can supplant or complement the automated processes currently found in most industries worldwide; these are the cyber-physical systems (CPS). These new elements are the fundamental part of recent smart enterprises. CPS are collaborating, computational entities which connect with the real physical world; the CPS get online information and process data using Internet services [3]. As systems comprising various technological elements, CPS requires knowledge of many disciplines such as software engineering, communication protocols, control theory, design strategies, and knowledge of novel areas such as edge computing [4]. The literature reports several methodologies, frameworks, and implementations of CPS. For instance, in [5], a cyber-application framework was proposed, advocating several patterns for partially handling ordered knowledge to build pervasive applications.

The CPS is comprised of assorted technological elements with different purposes according to the task assigned. In [6], the authors classified the elements of CPS from a production perspective: smart objects, CPS objects, and digital-twinning processes. Then, they proposed a systematic framework for the CPS, which includes two primary states: the interconnection of three physical segments and the communication of real physical and virtual devices.

**Figure 1:**
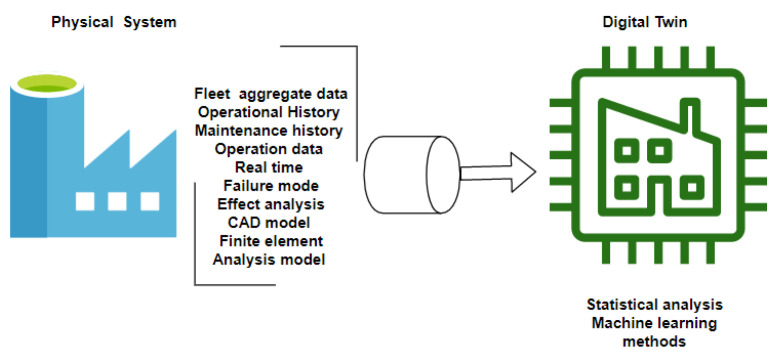
Digital Twin General Scheme.

Regarding the third element above, the Digital Twin (DT) is one of the fundamental parts of CPS. In recent years, DT has acquired relevance due to its usefulness in the industry. Several authors have also discussed the definition of this technological entity. The first definition is attributed to [6,7], which defined the DT generally as nonphysical elements described by digital models to represent physical elements’ behaviors in real-world environments [8]. With the experience and the effort of applying this conceptual technology, some authors added characteristics to the first definition. In [9], the authors discussed that DT must comprise five segments: the physical, virtual, connection, data, and service segment. This model was initially proposed by [10], whose authors argued that the five elements must have the same importance in developing any DT. The real-world device is the base for building the virtual segment; the virtual one allows to run the simulations, decision-making, and control of the physical device. The core of DT is the data form since it can allow generating new knowledge (Figure 1). Besides, DT can increment the complete performance of manufacturing systems.

Through innovative and effective tools such as DTs, developing new manufacturing processes and functions that collaborate to incorporate the newly emerging CPS into companies in their current state is conceivable. They can adapt these emerging systems in processes that already have a certain degree of automation.

Hence, the integration of standards to enable the communication between different hierarchical levels of manufacturing processes is a standard best practice in the industry. For instance, the international standard ANSI/ISA 95 usually facilitates integrating enterprises’ functions and the control systems through models and terminology that determine the information exchanged among different manufacturing operation levels [11]. Moreover, by integrating new CPS into the industrial processes, the standards for manufacturing execution processes (such as ISA-95) must be configured to incorporate new smart technologies and communication protocols.

**Figure 2:**
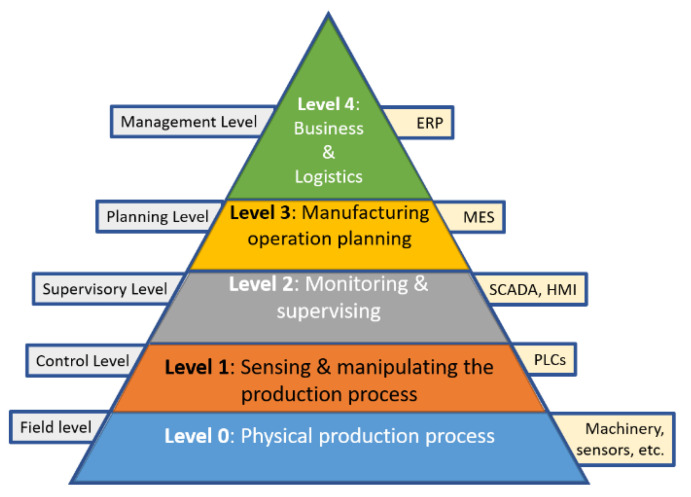
Automation Pyramid according to ISA 95 model.

The Automation Pyramid (AP) derives from the standard ANSI/ISA 95. This conceptual representation suggests the levels defined in the standard ISA-95 (Figure 2). Some efforts to update the AP through the concept of digital twin have been made. In [10], a shop floor virtualization case was proposed. This work explored the digitalization of the four main shop-floor components: physical, virtual, service, and data shop-floor systems.

On the other hand, data management in the manufacturing process must be considered relevant in the transition towards process virtualization.

A data-driven and model-based conception of a digital twin was presented in [12]. The model, in combination with machine learning techniques, aimed to achieve quicker improvement of manufacturing systems. Data management in the new architectures of manufacturing processes is crucial for achieving a smart factory’s transition. Therefore, some research works have focused on proposing the most appropriate models and algorithms for handling the large amount of data obtained from the CPS. In [13], the authors highlighted data properties such as data volume, variety, and critical selection to handle the information with certainty. Also, different works concentrate their efforts on applying intelligent algorithms to streamline the process to handle large amounts of data. Undoubtedly, artificial intelligence (AI) algorithms are the resource most suitable to manage data accurately. In [14], the machine learning (ML) applications in the industry are extensively discussed. The authors presented a broad overview of the relevance of ML in industrial applications.

From a manufacturing perspective, some authors have digitalized AP decision-making levels. For example, in [15], the paper focused on the practical implementation of a digital twin concept in a Manufacturing Execution System (MES). The proposed digital twin was built to monitor energy consumption inside a laboratory dedicated to Industry 4.0. Also, some authors have proposed modifying the architecture of MES from a static state to a flexible, dynamic information model. In [16], the proposal focused on the case-based approach to information modeling to generate new manufacturing systems that can integrate new flexible materials models. Thus, a link between classical MES and Enterprise Resource Management (ERM) is shown. However, the novel modeling was designed using the Reference Architecture Models applied to Industry 4.0 (RAMI4.0).

Currently, the manufacturing industry tends to incorporate smarter elements into more sophisticated systems. Thus, manufacturing begins to have more efficient, optimized processes with better communication and connectivity, which provides openness to real-time decision-making at any stage of the production chain. The interactions of technological elements and human capital have been increasing.

Therefore, current manufacturing models must evolve with new proposals incorporating elements that provide decision-makers with a more precise overview that helps them determine the direction of a product in each production stage. Consequently, the relevance of improving manufacturing through virtualizing the processes that comprise it is evident. Unfortunately, the current proposals only consider virtualization in some of the levels that comprise the current AP. As noted in the transition to smarter manufacturing, the free and sufficient flow of information among its levels is crucial, so the virtualization of just one stage is unwise. Consequently, this work conceptualizes a new complete model of the automation pyramid based on the digital twin concept. In addition to being a valuable tool in the decision-making process, total virtualization can facilitate the flow of information among its different levels under a single platform.

Also, this paper shows a case study of a DT applied to an educational environment. The characteristics of the proposed model are identified in the DT addressed. Augmented Reality (AR) and Virtual reality (VR) are discussed as part of DT development as emerging technologies.

## 2. Digital Twin General Concept

The DT description was shown in 2003 at the University of Michigan by Grieves in a PLM presentation [10]. Over time, several authors added their perspective to define the digital twin (DT) concept [17,18]. In [19], the authors defined the DT as a king of ultra-high-fidelity simulation, including a health management system, a historical vehicle, and swift data. However, the definition of DT was described in different manners, so the DT definition was not unified. One of the most frequent and accepted definitions of DT was presented in 2012 by [20], where the DT was defined as an integrated, multi-physics, multi-scale, probabilistic simulation of a complex product using the most suitable physical models to emulate the life of its related twin. In [21], it was mentioned that the DT is composed of a physical product, a virtual product, and the connected data that links both products. Also, the authors defined some relevant characteristics of DT:

(1) The physical and virtual worlds are combined and interchange information based on virtual models with high fidelity.
(2) Interaction and convergence: (a) in physical space, (b) between historical and real-time data, and (c) between physical and virtual space. Related to (a), DT integrates full-flow, full-element, and full-service characteristics, then, the data generated in the physical-space phases can connect. Regarding (b), the DT data requires expert knowledge and accumulates data from deployed real-time systems. Finally, the physical and virtual spaces are smoothly connected, and they interact easily.
(3) DT can update data in real-time so that virtual models can tolerate continuous upgrading by relating virtual space with physical space in parallel.

In Figure 1, the Digital Twin (DT) concept is illustrated. Some authors consider that this representation highlights the essential characteristics that a DT must possess [22]. However, some others have considered that a complete DT model should have essentially five elements in its structure: physical part, virtual part, connection, data, and service [10]. Some authors propose theoretical frameworks which consider the mentioned elements incorporating information processing layers [18].

By its definition, a DT could be a quintessential cyber-physical system when IoT is included as well as cloud computing, according to [22,23,24]. In [6], the authors proposed a systematic framework for cyber-physical production systems (CPPS). Its processing involves two situations: (1) the interconnection of three physical sections; (2) the mapping and collaboration of physical sections with virtual ones. The application in the manufacturing of DTs is a constant evolution; therefore, the proposal of DT application has different perspectives. In [25], the authors presented a variety of applications in manufacturing environments for the DT. The information, representing the authors’ perspective, is presented in four sections: DT-based production design, smart manufacturing in DT workshop/factory, product DT for usage monitoring, and DT as an enabler for smart MRO.

Some proposals focused their efforts on the theoretical foundation for the development of DTs. For example, in [9], the research for developing DTs comprises mainly four parts: DT modeling, simulation and VV&A (Verification, Validation, and Accreditation), data fusion, and interactive collaboration and service. As relevant information, the work presents an interesting review of DT industrial applications, specifically DT-related patents and DT applications by industrial leaders.

On the other hand, most of the DTs applied in the industry are closely related to the levels below the automation pyramid (AP) [10]. In [17], the authors proposed a framework of DT-based smart production management and control; then, the authors addressed the main contribution of this work: the prediction services for a satellite assembly shop-floor, using digital twin and Big Data technologies. The case study demonstrated how to apply the proposed framework on a satellite assembly shop floor. Equally, in [21], they proposed a framework for product design, manufacturing, and service-driven by a DT. The authors concluded with discussions of problem solutions about data in the product lifecycle, a new method for digital-twin-driven product design, manufacturing, and service, and, finally, the detailed application methods and framework of digital-twin-driven product design manufacturing and service.

Undoubtedly, the data plays a crucial role in the application of DTs. Therefore, theoretical proposals and applied knowledge of the management of data within DT are quite valuable. In [26], the authors addressed representative applications focusing on aligning with their proposed reference model. They presented a general data processing framework for constructing DT. It comprises data acquisition and cleansing, data storage, and time-sensitive data processing. The intrinsic relationship of data with the ERP and MES systems must be considered one of the main factors in the DT concept and its application. The research in [16] focuses on transforming the statistical architecture of MES to flexible and dynamic information models. The main contribution is the case-based presentation of a new approach for information modeling dedicated to new manufacturing systems. The shown models were deployed on the manufacturing system of smart electronic devices production, so they focused on several representative limitations in the previous generation of industrial computer systems.

Indeed, the presence of DT will be more constant and have a greater impact on applications within the industry. A well-structured theoretical base, standards for the regulation of its application, and its correct implementation per se, are part of the work that employees of the industry and the academic community must seek and continue to improve. The correct evolution and application of technological elements in the industry will be essential for transforming industrial life. This work seeks to be an innovative and supportive proposal for the industrial community, relating a technological element developed in and for the industry to educate human resources to be realistically prepared for their future work.

## 3. The ISA 95 Standard and Automation Pyramid Concept

This enterprise-control system integration was developed by the International Society of Automation (ISA) [27]. When a consistent and standard communication structure is required for integrating enterprise-control systems, the ANSI/ISA 95 could be implemented. ISA-95 also allows selecting the information transmitted and received between systems. Besides, the complete manufacturing system could be improved [3]. Thus, the ISA 95 standard is a guide to determine the requirements, select manufacturing execution systems (MES) suppliers, and lead the development of MES and databases [28].

As depicted in Figure 2, the ISA 95 standard proposes the enterprise manufacturing operation in five levels.

The zero level consists of the industrial process per se, the machinery, and the needed human resources.

The subsequent level consists of the automation components: the interaction between the physical components and the more basic control elements such as PLCs and their peripherals, sensors, and actuators in general. This level is mentioned as the industry hardware where the top-level systems’ interaction and the process occur. The equipment can be monitored through a human-machine interface (HMI) or supervisory control and data acquisition systems (SCADAs) at the monitoring and supervision level. Level three controls the manufacturing operations through manufacturing execution systems (MES), which control what processes should be executed and their order. At the top level, the financial, accounting, and marketing programs reside. The Enterprise Resources Planning systems (ERP) usually manage the inventory, billing, accounting, and logistics. This tool supports the accounting for company billings, expenses, and inventory.

The ISA-95 standard is based on models through which the interfaces, business systems, and manufacturing control systems are defined. The standard address benefits such as [27]:

- The time required for achieving the total production is reduced
- Correct tools for integrating control systems in enterprises are included
- A better and fasts manner of determining users’ needs
- The cost of the automated manufacturing process is reduced
- Optimizing supply chains
- Reducing lifecycle.

The operational models clarify the application functionality about how the information must be used within the different hierarchical levels. The standard determines which information must be exchanged within systems for sales, finance, logistics, maintenance systems, production, and quality areas. The UML models are the basis for the development of interfaces between ERP systems and MES.

In the literature, the primary efforts focus on developing proposals for optimizing and applying conceptually and practically the DT concept to the elements located in the first three levels. However, it is relevant for this work to discuss the functions executed by the planning and management levels, MES and ERP systems, respectively.

### 3.1. Enterprise Resource Planning (ERP) Systems

The enterprise resources must be incorporated into an information system efficiently to deploy Enterprise resource planning (ERP). This program coordinates the complete process and collects data analyzed and managed in different areas [29].

Also, in [29], the authors address some benefits of the ERP systems to Industry 4.0 (I4.0) implementations:

- A real-time data is monitored and examined so atypical situations could be detected
- A set of rules for business can be incorporated into the ERP systems to create an autonomous system
- Portable devices are integrated into the ERP to set a communication channel between the manager and manufacturing machines
- A well-organized online operation system could be set
- The orders’ information of orders can be tracked, and the status is updated

Some interesting research about ERP systems is presented in the recent literature. Some of these are about implementing ERP systems and integrating new technologies, such as cloud systems, into the traditional ERP systems [30]. While some authors conceptually integrate new emerging technologies, some proposals aim to implement cloud computing and ERP systems [31]. Compared to a single ERP implementation, the authors found that a hybrid solution is more suitable for interactions with the user. Their methodology undoubtedly opens the way to I4.0 technological integration.

The ERP system allows transactions to be managed by the business in the company using a shared, robust database with standard procedures and data exchange between functional areas [32]. In a wide range of production companies, ERP systems support production and distribution. These are implemented to partially integrate and automate financial, resource management, manufacturing, and sales functions [33].

The personalization of the systems is a trend, especially among medium-sized companies, so ERP plans are offered as a service through providers. Thus, new ways of providing the software are investigated, mainly related to cloud system hosting [34]. Nowadays, accessibility to remote devices is a critical feature of personal and industry devices. Remote access to information verification for decision-making is crucial for optimizing manufacturing processes. That is why ERP system providers face these challenges by offering mobile-capable ERP solutions [35].

**Figure 3:**
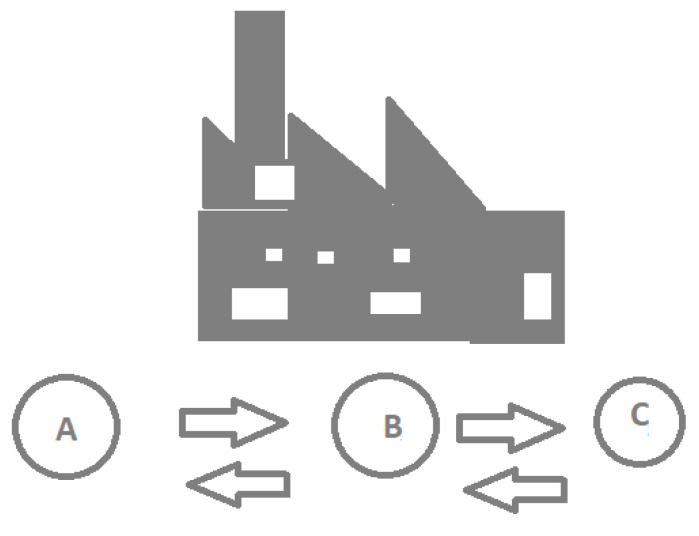
Functional enterprise/control (A—Order processing, B—Production Scheduling and C—Production control)

Standard ISA-95 content allocates specific functions to the various levels of business enterprise management (ERP) systems. Some of these activities are summarized in Figure 2. As it shows, information flows between the functions that cross the enterprise/control interface. According to the standard, a “function” is a group of tasks classified as having a common objective. These are organized hierarchically and identified with a name and number. The number identifies the data model hierarchy level. The Figure 3 illustrates the boundary of the enterprise/control interface [27].

**Figure 4:**
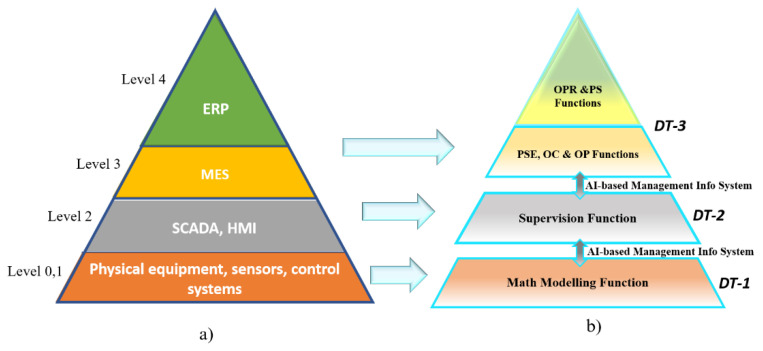
Automation Pyramid: (**a**) Traditional AP and (**b**) Proposed DT-based Automation Pyramid.

The general model represented in Figure 3 depicts an organizational structure based on functions. Thus, this work proposes a Digital Twin structure using functions as represented in Figure 3. As a result, in Table 1 are presented functions that correspond to those accomplished in the ERP and MES systems, on which the DT model is based, as shown in Figure 4.

Order processing (OPR)

The tasks assigned to this function are:

- Customer order handling, acceptance, and confirmation.
- Sales are estimating.
- Waiver and reservation handling.
- Gross margin reporting.
- Determining production orders.

Production scheduling (PS)

Functions interact with functions in the production control system through a production schedule based on actual production information and production capacity information—the functions and information created or adapted by the production planning. The production scheduling functions are listed in Table 1.

Production control (PC)

The fundamental functions of product control are shown below.

- Raw materials that have to change into the final product.
- Updating of process plans.
- Plant engineering.
- Raw materials’ requirements.
- Generate the reports for performance and costs.
- Assessing restrictions on capacity and quality.
- Self-test and diagnose production and control equipment.
- Create production standards and directions for SOPs (Standard Operating Procedures), recipes, and equipment management for defined processing equipment.

### 3.2. Manufacturing Execution Systems (MES)

Manufacturing execution systems (MES) were developed in the early 1990s as a specific application at the shop level. These systems mainly consist of production management and control functions such as scheduling, product quality management, control, production cost control, etc. This software aims to realize integrated production management and control on the shop floor [6].

According to ANSI/ISA-95, MES can link the office ERP systems to the shop-floor equipment by employing operational manufacturing management (MOM) functions in the factories [36]. Some authors include online characteristics in the MES definition, adding the MES accumulation of methods and tools to accomplish production, where the online characteristics are used as a connecting feature [37].

MES are the essential elements in the fourth industrial revolution (I4.0). MES can cooperate between complex tasks that associate several tools and procedures to accomplish the resulting collaboration. Owing to the cooperation of different IT systems inside and outside the enterprise, the MES must have open and flexible features. These include:

(i) uninterrupted communication with the production level,
(ii) conversion of information models online,
(iii) transformation of data from the business model to data that can be used in the in-dustrial control systems and
(iv) the conversion and processing of the data from the control systems into data that can be understood by different enterprise systems [16].

Nowadays, there are some improving areas in the implementation process of MES. The cost of commercial MES is high; besides, it needs an additional enterprise resource planning (ERP) system. Thus, it is a problem for small manufacturing enterprises (SMEs). On the other hand, the implementation of MES and ERP systems could need specialized hardware. Data analytics is not included for individual employees [38], so the current MES are implemented in large enterprises instead of customizable short-run products produced by SMEs [36]. I4.0 tends to customize the products, which seems not to be compatible with the ideology. Thus, problems identified in the current MES are generated by nonautomatic data entry, nonreal-time capability, interoperability problems, and data sharing among different elements of the MES [39]. Therefore, some proposals have addressed the characteristics of an MES to ensure compatibility with I4.0, including decentralization, mobility, connectivity, and integration on the cloud [40].

Despite the evolution of business and resource management systems, the connection between the process control devices and MES appears missing whenever an MES is applied. This gap reproduces that the two areas evolved unconnectedly and addressed different requirements regarding application context or data characteristics. Furthermore, there is evidence in the industry that different networking concepts also separate these two worlds. For example, LANs are fundamentals in offices, while the control level is the domain of field buses. Hence, the approach links two different network types and the interconnection of two approaches [41,42].

In the beginning, the purposes of MES were focused on finite operational scheduling, resource production management, and dispatching the production units. MES would also supply automated data collection and deliver detailed documents to the workstation. The MES manages uninterruptedly, up-to-the-minute, and on the spot [32].

## 4. Automation Pyramid and Digital Twin

The proposed model elements are defined in detail. As presented in the introduction, several efforts have been made to introduce DT schemes in different levels of the automation pyramid (AP); however, there have been no proposals applying the DT concept to the whole ISA 95 structure. The main reason is that the MES and ERP systems are essentially digital. However, their gaps and improvement areas discussed in previous sections are enough to justify seeking new alternatives in management systems in the industrial environment. Thus, aiming to attend to the gaps found in the literature closely related to real applications in industry, this work seeks to overcome the exposed weaknesses through an innovative tool such as the DT concept.

By making the entire AP model virtual through a simulation, the information flow through the stages should be automatically managed through an artificial intelligence algorithm. As shown, this flow would be bidirectional between the levels represented by three DT-based virtual models. Besides, with the entire pyramid virtual, the efficiency of the simulated process can be improved by running a massive quantity of simulations in a shorter time, which would be difficult to achieve with the one-stage pyramid virtualization. Then, the automation pyramid model based on DT is discussed below, and its general configuration is shown in Figure 4.

The virtualized AP model is made up of 3 DT models. Each DT is interconnected using the automation pyramid structure having and an AI-based management information system. The low level is based on math modeling functions; this DT integrates a virtual model that accurately represents the physical structure.

Besides, as an initial phase, it is proposed that DT-3 can execute the ERP and MES functions presented at the beginning of this work (OPR, PS, and PSE, OC, OP). Meanwhile, the role currently assigned to DT-2 will be supervisory. This function is one of the main characteristics of the traditional level-2 systems such as SCADA and HMI. For DT-1, the mathematical modeling function is assigned, essentially representing any element at level 0-1 that different simulation software such as Matlab and LabVIEW performs. As a result, the complete representation of the DT-based Automation Pyramid could be defined by DT-1, DT-2, and DT-3.

### 4.1. AI-Based Management Information System (AI-MIS)

At this time, artificial intelligence has been demonstrated to be a convenient problem-solving tool for decision-making. Besides, applying this type of mathematical algorithms for handling large amounts of data opens the gap for recent machine learning algorithms.

The amount of information generated in the modern manufacturing process is challenging to analyze and manage. Besides, this information could be used for predicting or improving the manufacturing process. Thus, understanding information efficiently is a fundamental step in the automation industry since several sensors integrated into IoT can send videos, pictures, audios, and so on [14]. Moreover, the generated data can be easily deployed and used. The performance of manufacturing systems could be incremented as the decision-making system performance is improved and the cooperative working. Thus, an agile manufacturing process can be implemented. When agile manufacturing based on management information systems is implemented, the partner cooperation is incremented. The manufacturing industry is a dynamic virtual enterprise that can quickly respond to market demands [22,43].

**Figure 5:**
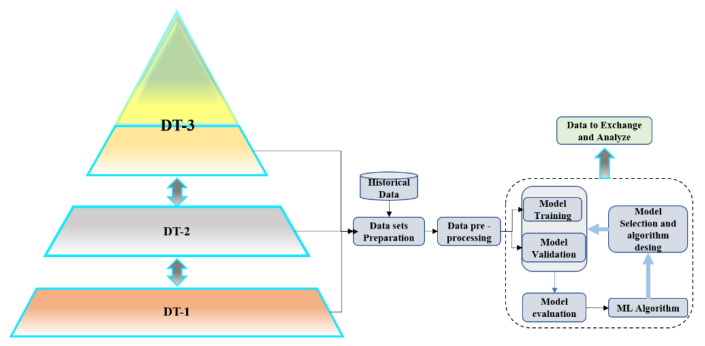
Proposed AI-MIS.

Thus, the interactions among the different DTs generate a large amount of various data. So the exchange of information, the selection of data types that should be exchanged, the weighting of transition times, and the route to the DT level must be managed efficiently and safely. For this reason, the proposed model must have an information management system based on machine learning, given the nature of identification, classification, and prediction of these AI algorithms. In Figure 5, the proposed model for the AI-MIS is shown.

The management system model for information management among DTs is based on a presented model [14]. The data set preparation would involve extracting the data from a historical and real-time database, examining this structure, and selecting through a sample and variable directions. The data pre-processing usually helps to improve the data quality by making data transformations. These shifts are related to fitting the time axis data together to avoid inconsistencies in the essential variable. Also, the outliers have to be eliminated from the dataset. In addition, some missing relevant data need to be addressed, and finally, the scale has to be adjusted according to the process variables. Once the training data set is prepared, the ML algorithm is selected according to the data characteristics (different within DTs), the complexity of the data model, etc. The ML algorithm between DTs would be different; the selection would be based on different criteria (Akaike Information Criterion, Bayesian Information Criterion, and the Hannan–Quinn Criterion) [44].

The proposed data analytics model is intended to be used either offline or online according to the functions assigned to each DT.

### 4.2. Field and Control Level Model

**Figure 6:**
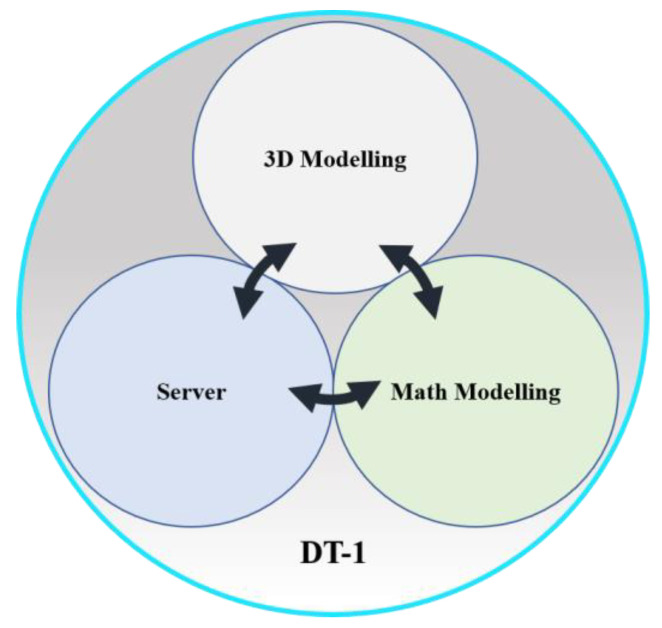
DT-1 Virtual Model.

Mathematical models commonly describe the physical elements contained in the field and control levels. These are usually simulated in dedicated software such as MATLAB and LabVIEW. This work precisely proposes the mathematical simulation of actuators, sensors, and control systems to be developed in any of these platforms. To be correctly synchronized with the rest of the levels, these simulations must be developed with specialized hardware that supports real-time simulations. Usually, this kind of hardware is based on field-programable gate arrays (FPGAs). This characteristic enables the proper communication of all the systems integrating the virtual pyramid. Thus, the initial two levels are proposed to be virtually modeled in the dedicated software mentioned and executed in a real-time environment. Figure 6 shows the DT-1.

The virtual representation of the first levels of the original pyramid will be designed in addition to the 3-D mathematical modeling. The representation of mechanical devices in 3D models is essential for user interaction. Through CAD tools, it is possible to have a virtual representation of the physical models of any machine. Currently, CAD modeling software allows its platforms to interact with mathematical modeling software such as LabVIEW or MATLAB. A server is required to interact with the virtual model to access the DT remotely.

The data obtained from DT-1 can be extracted to analyze and apply algorithms that contribute to maintenance prediction, raw material monitoring, and production analysis. It can be available for any of the rest of the virtual models.

### 4.3. Supervisory Level

The traditional pyramid automation considers SCADA and HIM systems to supervise the production levels. To simulate this characteristic, this work proposes to replace these supervisory systems with virtual vision-based monitoring systems. Hence, the proposed function of supervision will be simulated in a virtual environment by the DT-2.

**Figure 7:**
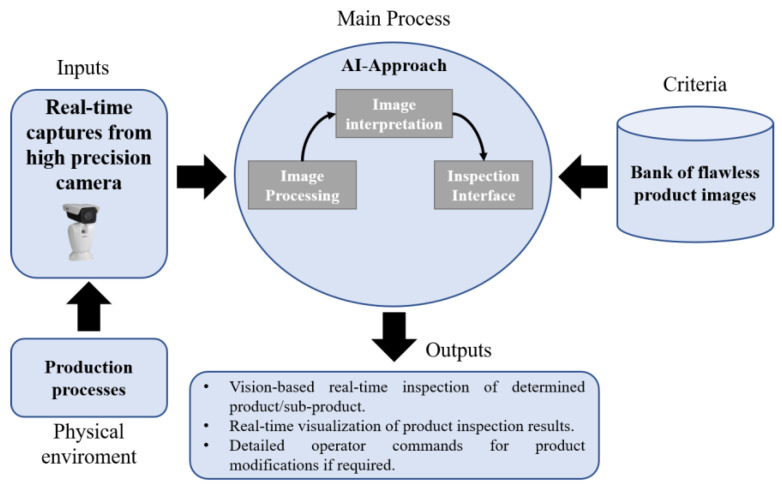
DT-2 Virtual Model.

Currently, in industry, the supervision of production through image analysis is widely studied. Techniques for classifying and detecting failures based on artificial intelligence (AI) are usually employed to find anomalies in the production quality. Hence, the virtual supervisory level should use an AI-algorithm to classify previously captured images. Images with different characteristics would be stored in an image bank according to the production process. Later, these can be employed to classify those processed in real-time, captured by a high-precision camera. Figure 7 shows the DT-2 virtual model.

The images will be captured by the camera, whose handling software must be operating in real-time. The images, as shown in Figure 7, are extracted from the physical production process. In the image processing stage, the images have a preparation process improving their quality successfully in the interpretation stage. Frequently, real noisy environments can affect the computer vision algorithm because the lighting conditions could also change mechanical vibrations in the camera’s mechanism, etc. [45]. Therefore, to obtain an accurate result from the selected AI algorithm, the real images must be pre-processed to remove the image noise completely. For instance, ensuring mechanical circumstances such as proper mobility of the camera mount and adequate distance to the capture point can facilitate techniques such as a frame-difference approach [46] to remove image background.

For image representation, the pre-processed images are compared with those pre-selected and stored in the image bank. The images stored should be unique images with non-defective patterns. These images can be from a specific subsystem, subarea of the product, or a product full-view to detect wrong positions, for instance. The inspection interface must be a type of HMI where the AI results can be deployed. The AI outputs generally will involve the vision-based inspection of a determined product or product component. Also, if a faulty product/component is detected, the DT-model must deploy a pre-short report with immediate actions to be executed by the operators.

### 4.4. Planning and Management Level

As an initial proposal in this work, the planning and management levels are thought to be performed by AL-learning systems. These focus on the functions deployed by the ERP (order processing and production scheduling) and MES (the functions related to production control), as indicated in Figure 3. Decision-making systems, such as MES and ERP, can be considered highly nonlinear systems as they involve a considerable variety of scenarios and combinations due to the number of entrances, conditions, functions, and outputs with which they operate. Usually, AI-learning algorithms such as Multilayer Neural Networks (MNNs) are used as alternatives to learn linear and nonlinear relationships between input and output vectors.

**Figure 8:**
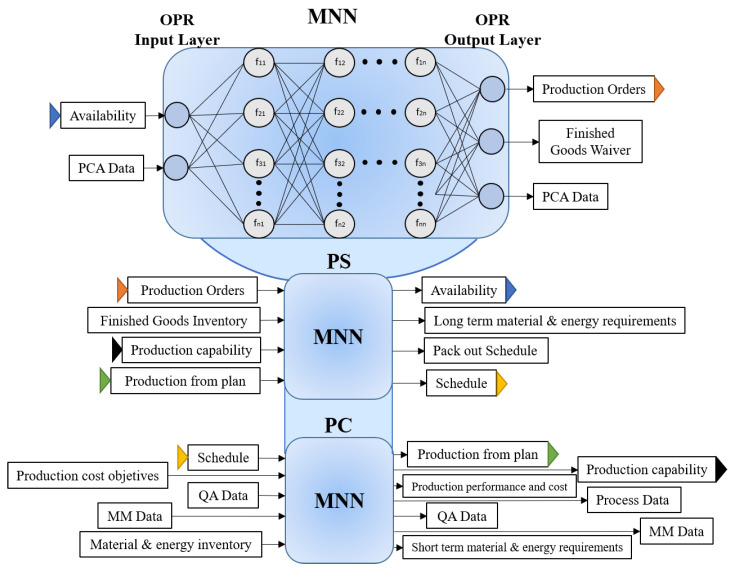
DT-3 Virtual Model.

Regarding using MNN, this method is based on primary interconnected neurons or nodes. The neuron receives signals from other neurons; the neurons’ inputs are affected by associated weights so that the output signal can be calculated as the output of an activation function. The input is the sum of the neuron’s inputs multiplied by the associated weights. The interconnection of neurons that are grouped into layers allows MNN to approximate nonlinear relationships [47]. Thus, the initial proposal is to use MNN to execute the functions and tasks already mentioned by the ERP and MES systems. Figure 8 shows the approach considering the MNN and the functions mentioned as part of the DT-3 virtual model. Figure 8 integrates a multilayer neural network in which the inputs are available and PCA Data; those inputs are represented by only one input neuron, but they are expanded to a cluster of neurons according to the required inputs. On the other hand, the outputs are production orders, finished goods waiver, and PCA Data; those outputs also have to be expended to accomplish the required outputs. The MNN only represents, in general terms, the input-output relationship, but it does not represent the total number of neurons required in the input and output layer.

The proposal consists of three MNNs that represent the three functions considered for this initial proposal. For practical graphical representation purposes, only the first MNN illustrates a general configuration of the MNN body. It is observed that the input and output layers contemplate the tasks performed by the order processing function. In this case, the OPR function has two input tasks and three output tasks. It is worth mentioning that referring to the name of a task involves all the possible values obtained from that specific task. Remember that an artificial neuronal network tries to model the behavior of a biological neural network. The network’s hidden layers will have a certain number of neurons, each associated with an activation function. In MNN, the body of each neuron represents a linear adder of external stimuli followed by a nonlinear activation function whose task is to use the sum of the stimuli to determine the output activity of the neuron [48].

Some of the inputs and outputs of each network are labeled as “data.” Regarding the PC network, the inputs and outputs with this label are shown for illustrative simplicity, since these tasks consist of two inputs and two outputs, i.e., the value of “QA Data” as input is two sets of values: “Standards and Customers” and “QA results,” according to the functional control model on which this DT is based. In the first OPR network, the PCA Data is the information shared between the PCA and OPR functions as determined in the standard. Accordingly, the input PCA Data value does not correspond to similar values to the output PCA data information. This relationship applies to the rest of the outputs associated with the PC network labeled “Data”.

On the other hand, some of the tasks in each of the OPR, PS, and PC networks have an arrow after or before the label with a particular color. Those with the same color indicate that the set of values derived from this task as output will be the same set of input values that some other of the two networks will receive. For instance, the PS network output data set for availability will be the same data set that the OPR network will receive as input. The graphic union between each network indicates that they are part of the same DT. The networks will have to be executed as a single system whose internal subsystems interact simultaneously, yielding partial and final results in real-time.

Consider the environment where the activities of the input layer are executed. The expected results are at the exit of the proposed networks. The main objective of applying the MNNs is to have an intelligent virtual system that can learn automatically; thus, the Backpropagation algorithm is proposed for MNN training. Backpropagation networks work under supervised learning, so there must be a set of training instructions that describe each output and its expected output value. To “train” the neural network, one must set a data set containing input signals connected with corresponding targets. In each iteration of the training process, the values of the weights are adjusted using new data from the dataset defined for training. Weight modifications are calculated using the backpropagation error algorithm for supervised training. Each step of the training begins by forcing the inputs out of the training set. Then, it is possible to determine the output values of the signals of each neuron in each layer of the network [48].

The DT-3 virtual model has as its initial main objective to emulate the decision-making carried out in typical ERP and MES systems, focusing (for now) on covering those discussed in the development of this work. Therefore, the DT-3 model can serve as an interface in which the complete detailed information of each task contemplated in real-time can be obtained. The DT-3 can be a valuable tool in simulating scenarios with real-world conditions and real-time responses or programmed responses to be displayed in a certain period. The DT-3 virtual model, with its proposed functions, can monitor production, provide information to control product batches, and label them for identification, information on material waste, machine downtime, and status-of-the-process data. This means the activities related to production control are particularly contemplated for the PC function and all those considered in the ISA-95 standard for the OPR and PS functions.

## 5. Case Study: Production System for Educational Purposes Using a Digital Twin Topology

**Figure 9:**
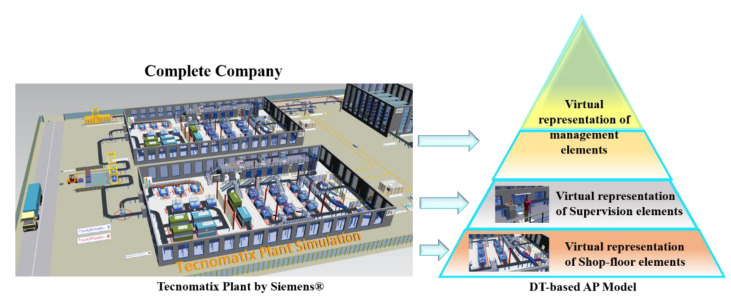
DT-base AP model for complete company application.

In the same way that the traditional pyramid can represent all the company levels, the DT model proposed in this work can be applied to a company as a whole, a manufacturing plant, a designated plant area, or a particular process (Figure 9). On the other hand, this case study is not considered a complete AI-MIS model since it is an academic application that illustrates the performance of the proposed structure. However, a classification model to detect defective based on a simple neuron is included to show the proposed framework using IA.

In the case study shown below, the application of the model is part of the development of DTs for workstations applied in the teaching of undergraduate students.

In situations of social isolation (COVID 19), such as today, having access to remote workspaces becomes crucial to day-to-day activities. The DT concept offers the opportunity to run systems remotely without personal presence in the workplace or classroom to manipulate the equipment. This case study applies the DT concept to remotely manipulate industrial equipment used as a learning tool for undergraduate students at Tecnologico de Monterrey in Mexico City.

For the teleoperated manufacturing system, some projects had been developed providing technological elements. In the current DT-based AP model, the Modular Production System (MPS) has elements that can be classified into the DT-1 and DT-2 models. Thus, an overview of this case study is presented first, followed by a detailed description of each element in the proposed DT-based AP model framework.

### 5.1. Description

The MPS belongs to the remote laboratory network of Tecnologico de Monterrey. The MPS objective is to develop technological distance education platforms that facilitate the transmission of information and knowledge and develop skills, abilities, and competencies obtained through experimentation and practice in real environments.

**Figure 10:**
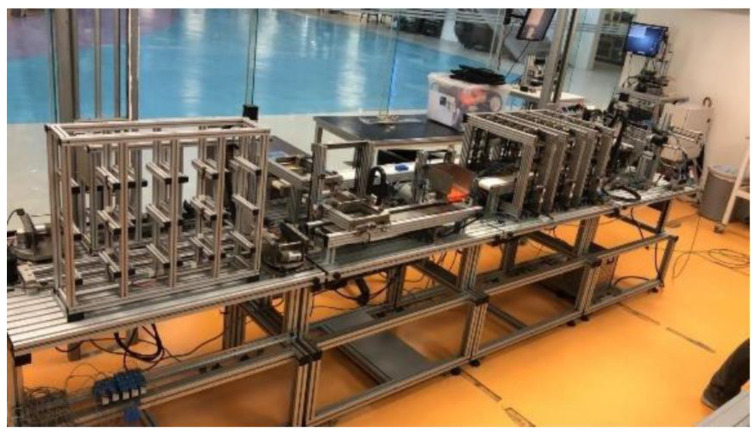
Physical Workstations.

The MPS has four workstations (Figure 10) that together represent a product line that can be configured, monitored, programmed, and remotely manipulated through a platform that manages access and system integrity.

This access system is a web application stored on a TEC System server, allowing users’ access through a section system; only the user who has reserved a time slot can use the MPS.

**Figure 11:**
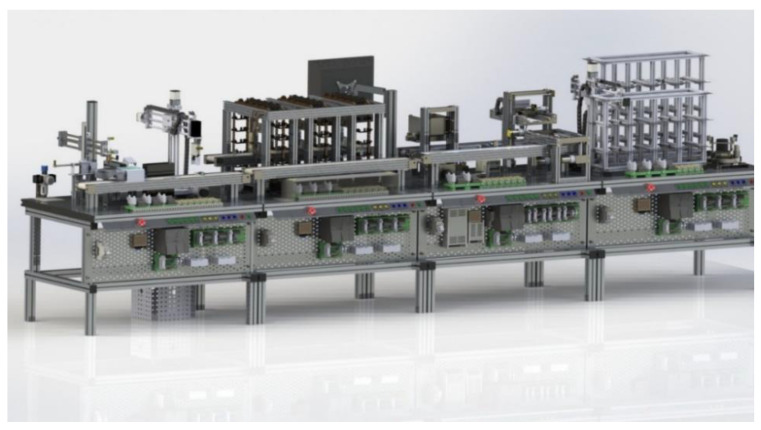
Complete Virtual Model of MPS.

The students access the virtual laboratory for uploading and configuring the physical system (Figure 11).

**Figure 12:**
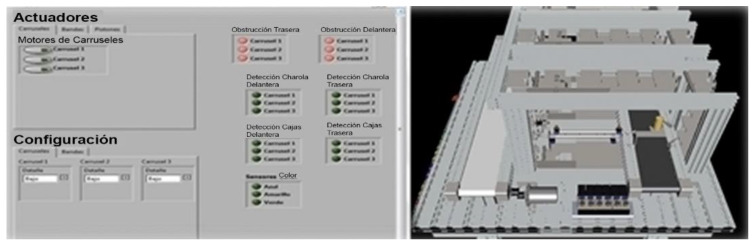
View of one module regarding the Virtual Laboratory Platform.

It has a three-dimensional computational model in which students can run experiments avoiding damages to the physical equipment; they can optimize their user time because the three-dimensional model can be accessed simultaneously by a limited number of users. Once the instructor validates that the students’ settings and programs do not have errors, the instructor generates an access key on an internet page so that students reserve a time slot in the remote lab to test their programs and settings (Figure 12).

**Figure 13:**
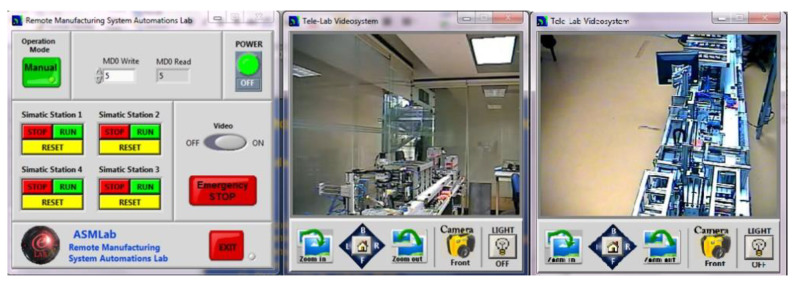
Application to access the Remote Laboratory.

To access the remote laboratory, students have to install an application specifically designed for MPS that allows them to work with the equipment remotely. They can also have visual information in real-time using video cameras (Figure 13).

### 5.2. The MPS in the Context of the DT-Based AP Model

DT-1: CAD Models and Server—The modular training system consists of four reconfigurable manufacturing stations which reproduce an automatic and modular production system.

**Figure 14:**
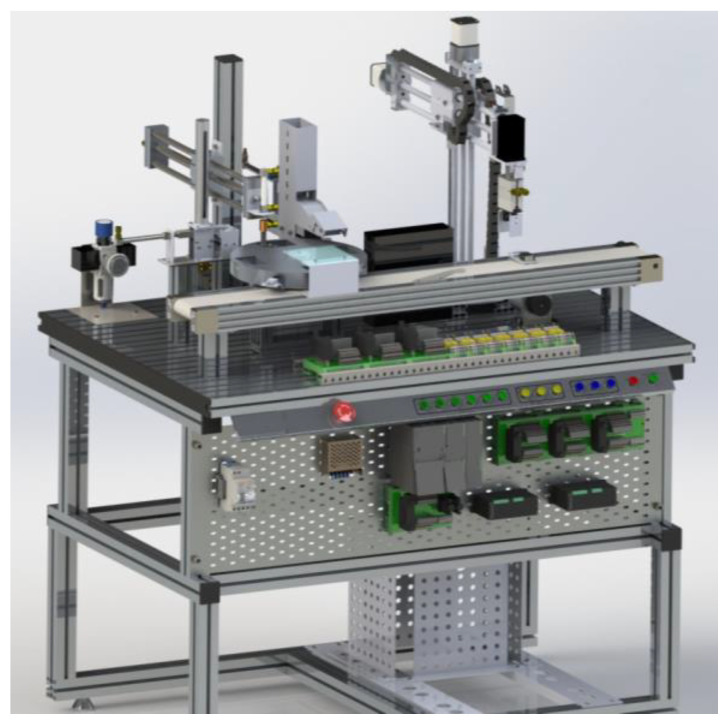
Virtual Model of Workstation 1.

Workstation 1—The MPS initiates this workstation for the production and circulation of materials. First, it receives raw materials (plastic boxes with two circular spaces and empty metal cylinders inside the boxes) to deliver the end-product boxes already armed with two metal cylinders (“armed with” refers to the cylinders in the boxes). Then, it moves the product already armed toward the end of a conveyor. Finally, a manipulating arm removes the assembled product from the input of the next workstation (Figure 14).

**Figure 15:**
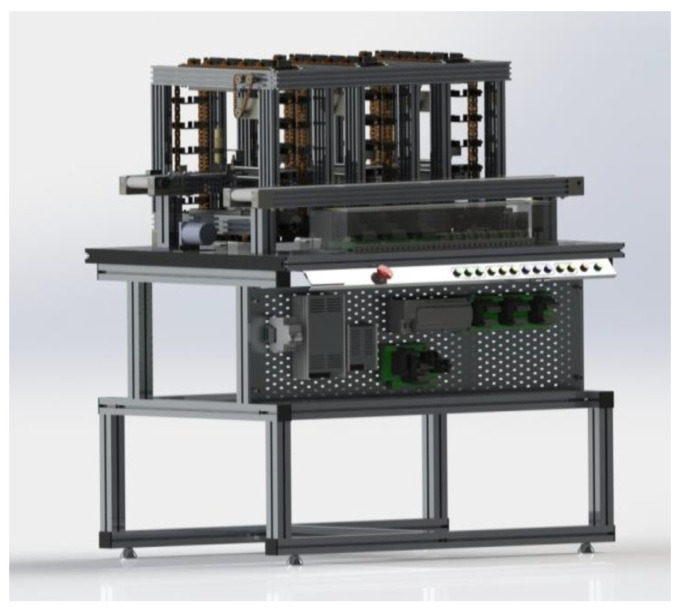
Virtual Model of Workstation 2.

Workstation 2—This station process begins when a loaded box (chips) is transported to the conveyor belt by the robot manipulator of Workstation 1. This is an automatic unit for sorting and temporary storage. Along the conveyor, path three sensors are arranged that identifies the color of the box. The sensors classify the cargo box to store it in one of the three vertical storages arranged along the conveyor belt (Figure 15).

The SCADA system counts the parts and sends signals to the workstation controller (PLC) to dispatch a certain number of boxes from the three storages to a second output conveyor belt that moves boxes to the table.

**Figure 16:**
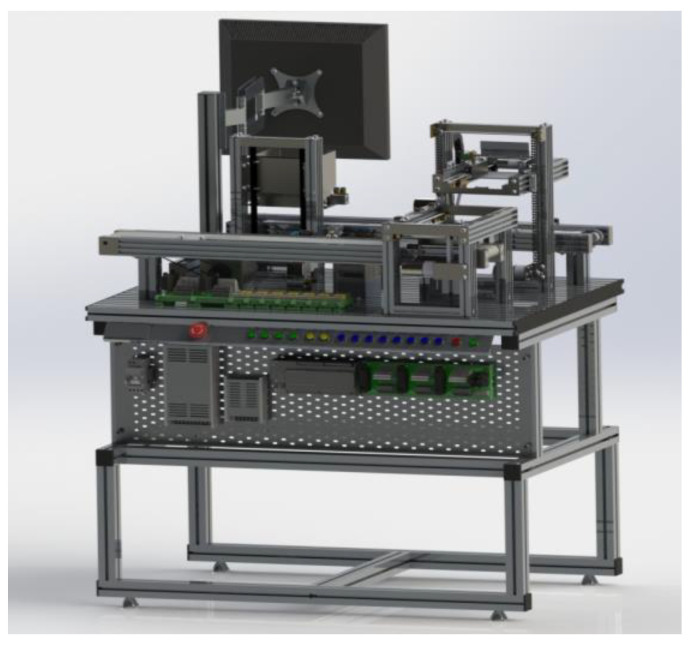
Virtual Model of Workstation 3.

Workstation 3—This workstation is the arranging and packing station. It receives the classified product of Workstation 2, then organizes it on the pallets to facilitate its transportation to the last workstation of the platform. Once the boxes are organized and armed on the pallets, they are placed on a chain that transports them to Workstation 4 (Figure 16).

**Figure 17:**
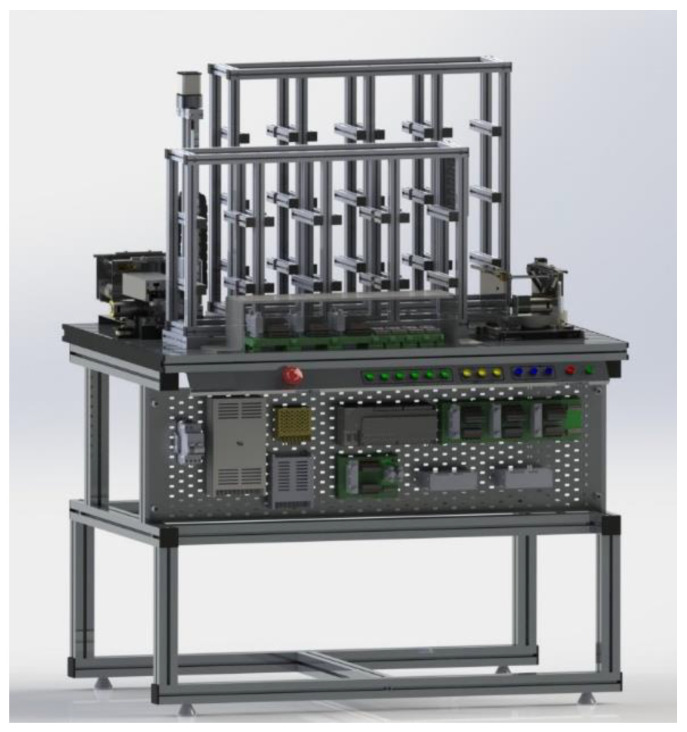
Virtual Model of Workstation 4.

Workstation 4—This storage and delivery station is responsible for storing the pallets delivered by Workstation 3 that contain the finished product in two vertical storages for subsequent release. The station consists of two vertical warehouses, two turntables, and a manipulator arm with three degrees of freedom (DOF) (Figure 17).

The Server—The system has an SQL server, which allows different operations and improves the encoding and orientation of objects. A single operation is equivalent to one or more programs in a low-level language. The server has an ASMLab platform designed to give students easy use of a remote lab. The platform allows monitoring of the MPS through cameras and microphones to supervise the process.

### 5.3. DT-2: Real-Time Manipulation Interface, Monitoring System, and Augmented and Virtual Reality

Currently, the teleoperated system has a SCADA system that performs the typical monitoring tasks of this system. Additionally, cameras have been placed to monitor the entire MPS process. For now, the monitoring process of the cameras is performed briefly, without using any AI algorithm to detect failures in the products or the production line. Before reaching this point, it was found that a priority should be a user interface allowing real-time communication of the SCADA system with the MPS. Thus, the solution for communication between SCADA and MPS is discussed below.

### 5.4. OPC Integration with LabVIEW

To achieve communication between LabVIEW and the PLCs in this project, it was necessary to use an OPC server. The OPC server allows declaring the shared variables used in the PLC programs and the interface for the SCADA.

Shared variables consist of Boolean memories, byte memories, counters, inputs, and outputs. The four workstations were created in the OPC panel to perform as clients that will communicate with the OPC server. Table 2 illustrates some of the shared variables between the LabVIEW interface and the OPC server for Workstation 1.

Similarly, the variables were declared into the four workstations using LabVIEW. Thus, it was possible to integrate any program, libraries, controls, and modified indicators into one single location.

### 5.5. Blocks in the Interface in LabVIEW

**Figure 18:**
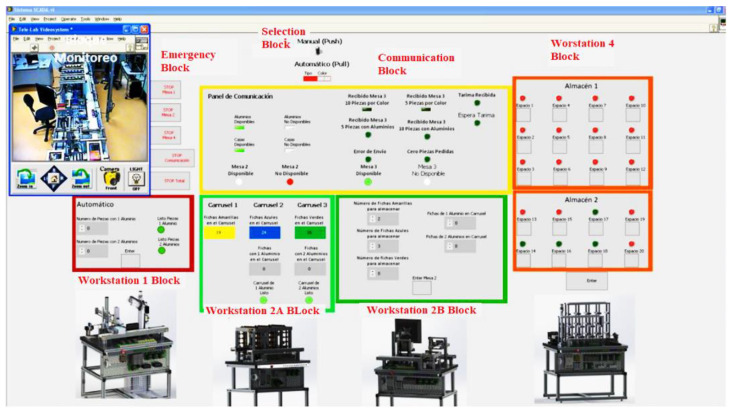
MPS Interface in LabVIEW.

Figure 18 shows the complete interface of the SCADA. A detailed description of each block is below.

Emergency Block—In this block, the user can stop the SCADA system process if an emergency or error occurs, stop a specific block, or completely stop the program from running.

Selection Block—In this block, the user selects how the system works manually (push) or automatically (pull). The production of the temporary and assembled storage depends only on the stock in the warehouse. Also, it is possible to select if the user needs a production based on the type of card or color.

Communication Block—A communication alert panel is implemented between the four modules, plus the status of critical sensors in the production process. The sensor’s status for aluminum cylinders and the sensor for plastic cases are shown on the right side of the box. The user will be able to identify the availability of material to produce. If there is no availability of material, the user must feed module 1 with the material.

On the lower left side of the block, two indicators show the status of Workstation 2. The user can detect in real-time if the module is busy performing any task, such as classifying a new card and identifying the status of availability of the carousels. In addition, to identify and correct physical anomalies, the upper right part is the communication alerts for module 4. For example, the user can observe when a platform has been received for storage or a different condition.

Also, the communication indicators for module 3 are displayed. For example, it can be seen whether 5 or 10 pieces have been received with aluminum or 5 or 10 pieces classified by color. Likewise, the user detects errors in the connection between modules 2 and 3 and order errors. The system only allows combinations of pieces whose add up are five or ten; any other combination is not processed.

Also, the user can visualize the availability of module 3 and observe its present status: available, stowing tokens, or transporting the tokens to module 4’s reception position.

Workstation Block 1—Here, the users can select the number of tokens they want to manufacture with one or two inserted aluminum. This option is only available if the manufacturing is done by type. Additionally, the program hides the keyboard when the production process runs to avoid overwriting values.

Workstation Block 2A—Station block 2A allows the user to view the status of the three module carousels. This block indicates the number of pieces in each carousel to verify the availability of pieces and empty or full carousels.

Also, two indicators report whether carousels 2 and 3 are in the process of storing parts by type. The indicators easily identify errors in the communication between the field devices and the SCADA system.

Workstation Block 2B—This allows the user to select the number of tokens to request module 2, with the restriction that the sum of the combination is 5 or 10. Both types and colors show the two controllers. The selection process will begin when the user selects the number of chips they want to accommodate on a pallet, followed by pressing the Enter button.

Workstation Block 4—In station block 4, the user can visualize the availability of the 20 warehouse spaces. If the indicator is red, then that place is unavailable; otherwise, the place is accessible.

Modular Production System Animation—The workstations were simulated in SolidWorks to be integrated into the human-machine interface designed in LabVIEW. This animation allows the operator to observe the functions of the stations virtually. The advantage of having this simulation is to simulate the process to detect improvements or failures.

The animation is controlled by LabVIEW and CAD files of the workstations integrated into the LabVIEW project. The workstation variables (inputs, outputs, sensors, etc.) found in the animation LabVIEW project are linked to the project’s shared variables that control the interface, making the MPS and the animation run synchronously. 

**Figure 19:**
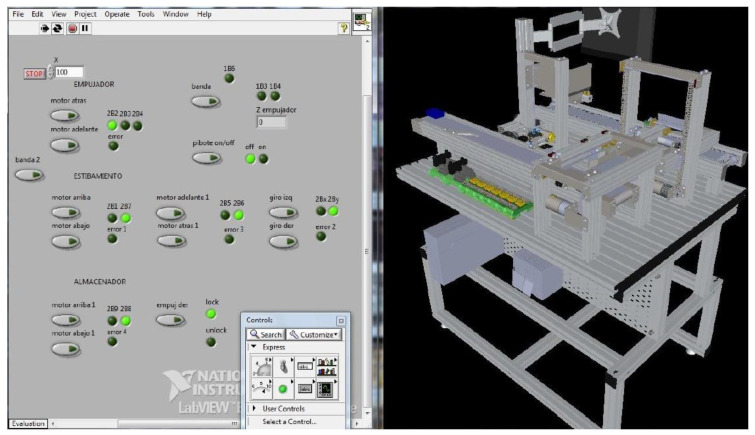
Workstation 3 Animation.

Taking Workstation 3 as an example, it was simulated the conveyor belt movement through a subproject carried out in LabVIEW. This action was repeated for all moving components such as boxes, platforms, and chains. Figure 19 depicts the indicators and controls used to simulate the movements of the station.

**Figure 20:**
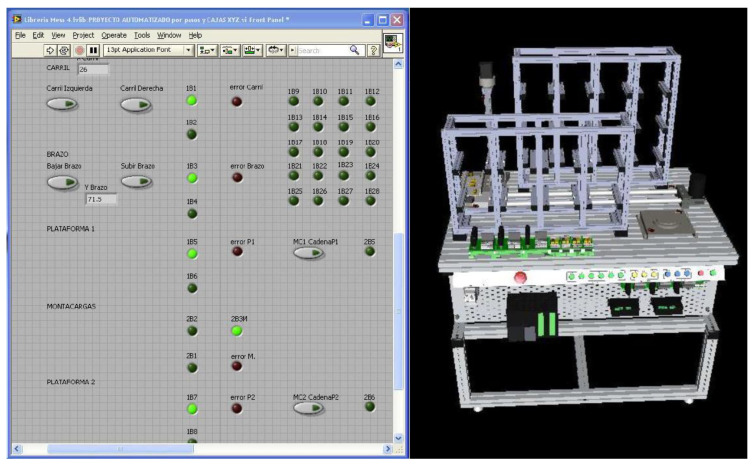
Workstation 4 Animation.

Similarly, Figure 20 displays the indicators and controllers for the variables of Workstation 4.

**Figure 21:**
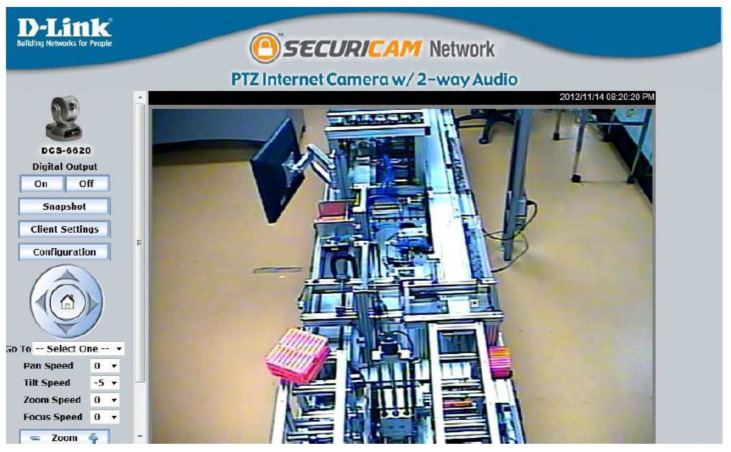
MPS view of monitoring camera.

Monitoring System—Regarding the monitoring system, security cameras allow the physical system to be monitored in real-time, mainly to prevent collision between components. The cameras are embedded in the ceiling of the laboratory. The cameras have an IP address that allows remote monitoring through the web (Figure 21).

The communication of all workstations could be done via ethernet through the SCADA system to ensure continuous production, control, and supervision. Now, the monitoring system is based on the LabVIEW environment. It is using an OPC system that allows incorporating new variables that are controlled.

In the MPS, the user currently has total control from the SCADA system to each station, and the total manipulation of production must be from the graphic screen.

Augmented Reality (AR)—The augmented reality (AR) application was made for Workstations 3 and 4. The objective was to generate an operation manual for these workstations through AR. The AR application was made with the Vuforia software, part of the PTC package, where it can be designed from two views, 2D and 3D.

**Figure 22:**
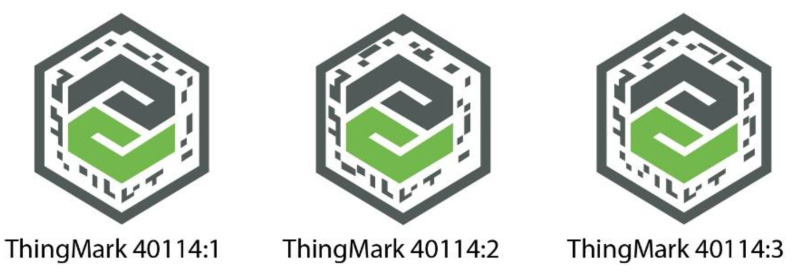
Thing mark codes in the AR application.

The AR application was observed through a Thingmark that works as a QR code. The Vuforia View application made it possible to scan and view the jobs hosted in that environment. Figure 22 shows the Thingmark codes.

**Figure 23:**
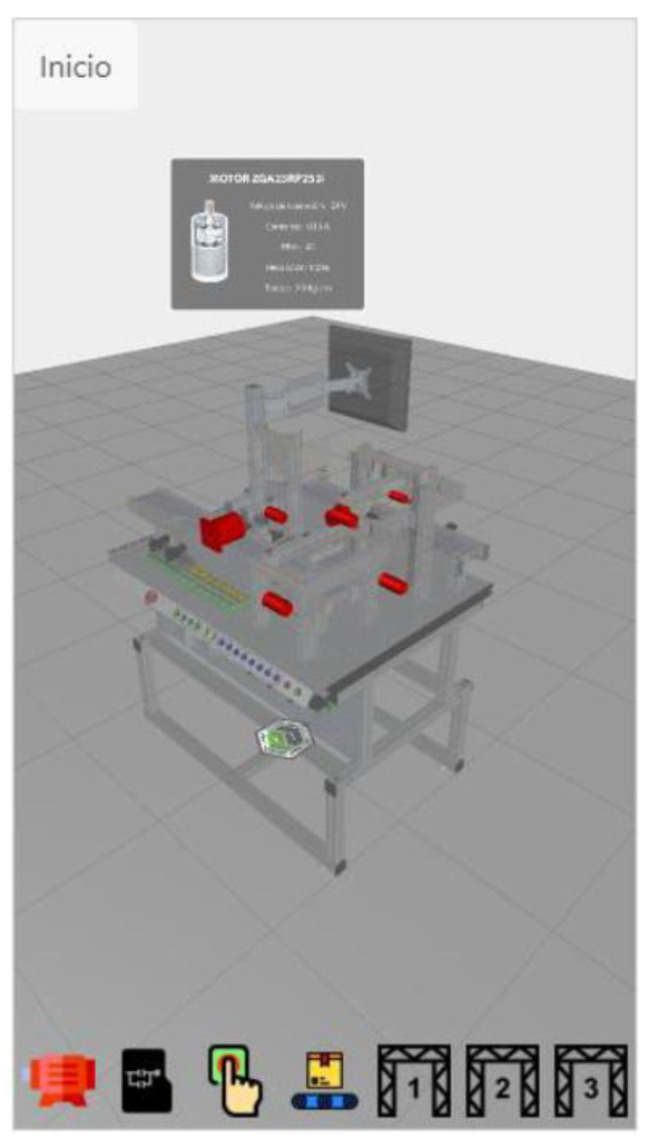
3D model interaction through Vuforia software.

Also, the application permits interacting with the 3D model as a single piece or with each element comprising the workstation. Each button has an assigned event in the application when pressed (see Figure 23).

**Figure 24:**
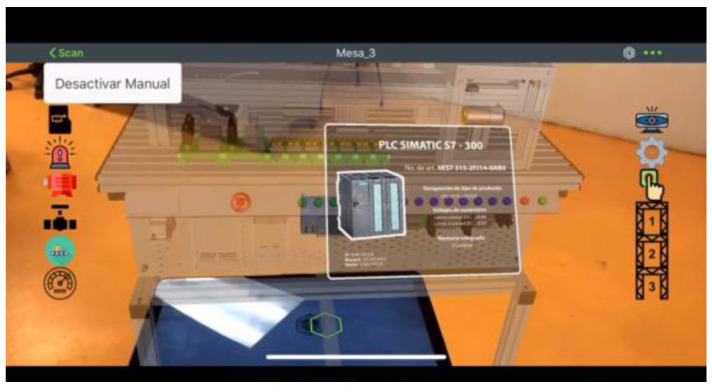
AR of Workstation 3.

Similarly, the workstations can be visualized using Augmented Reality glasses and the designed APP, see Figure 24 (AR for Workstations 3).

**Figure 25:**
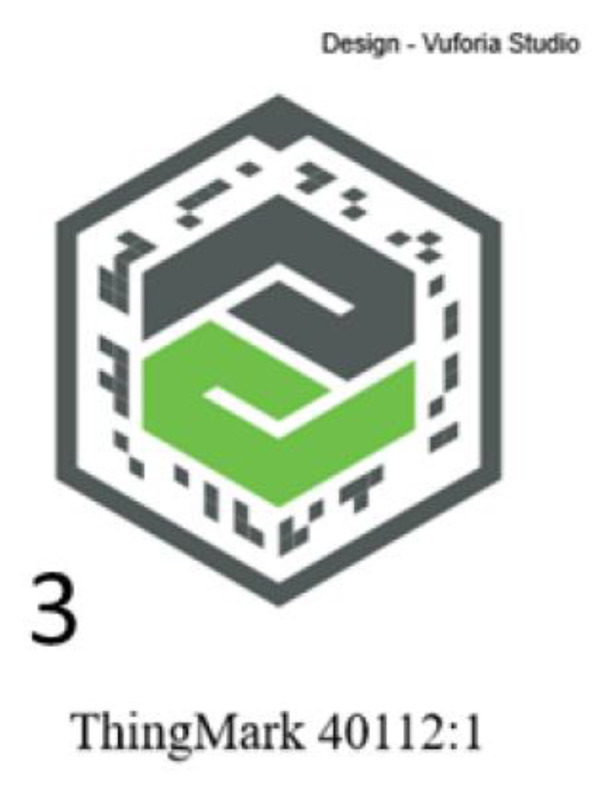
Thingmark for Workstation 3.

The buttons on the panel allow selecting an element from the table, which is indicated in a virtual model; a pop-up window will be displayed with relevant information on the selected element. The selection of the PLC is shown in Figure 24. One must scan the Thingmark in Figure 25 with the Vuforia View application to access the augmented reality model.

**Figure 26:**
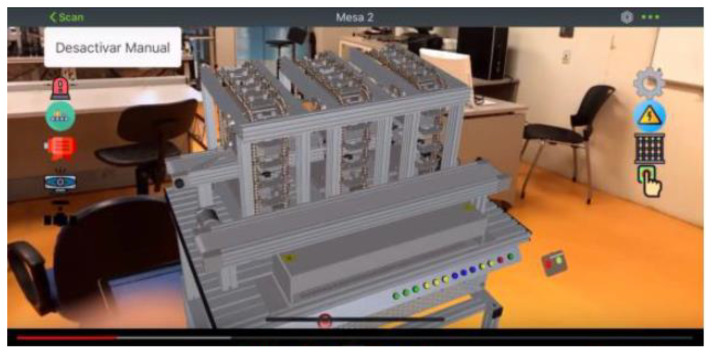
AR model of Workstation 2.

**Figure 27:**
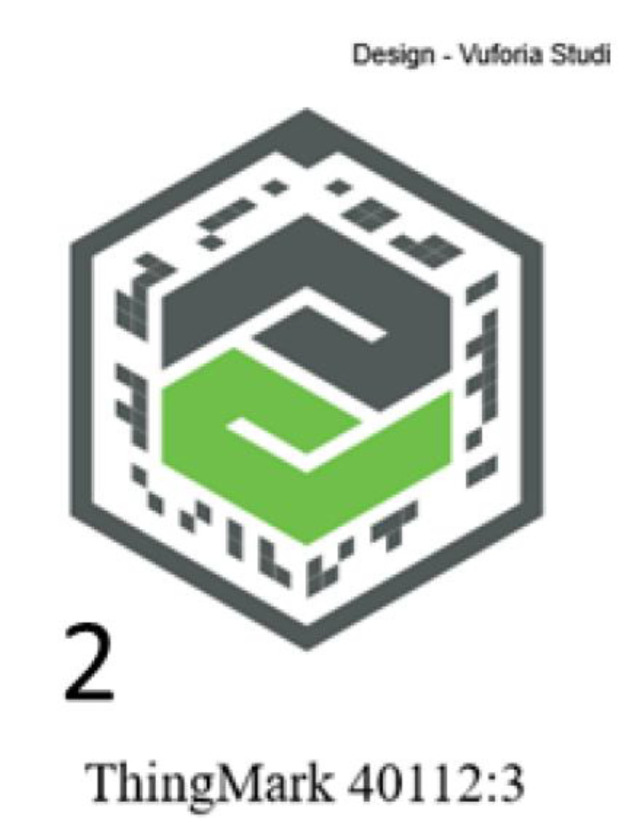
Thingmark for Workstation 2.

A second application allows accessing the AR model from Workstation 2. This is shown in Figure 26. Access to this model is done by scanning the Thingmark, as shown in Figure 27.

Virtual Reality (VR)—Through the virtual reality application, one can interact and monitor Workstations 3 and 4, Three Siemens products were used to make this application: Process Simulate, OPC Scout, and TIA Portal. With the latter’s help, the PLC of Workstation 3 was programmed, whose main task is palletizing.

The animation of the 3D model of Workstation 3 replicates its corresponding physical model (DT). The interaction between both models is carried out through a client and an OPC server, which obtains valuable information and data from the PLC. The information of available devices is giving by the OPC, and the OPC client links the server and accesses the offered data.

Process Simulate virtually validates and simulates the movements of each subsystem on the workstation. The interaction between this software and the variables declared for the PLC has a similar behavior of the virtual and physical systems in real-time. The OPC communication collaborates in a continuous flow of information without interruptions. The virtual identification of the output data under certain stimuli to the system’s input by actuators, switches, and sensors allows monitoring the system in real-time.

**Figure 28:**
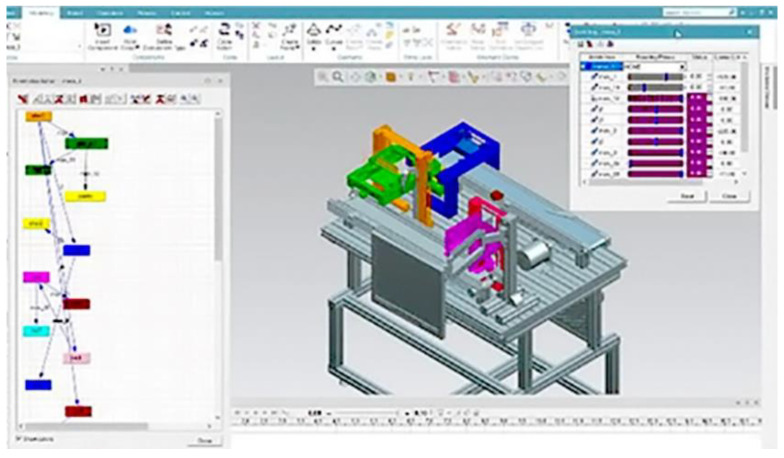
Process Simulate platform with the virtual model of DT Workstation 3.

On the other hand, through the Process Simulate platform, one can see the DT of Workstation 3. With this DT, one can manipulate and monitor Workstation 3 remotely (Figure 28).

### 5.6. DT-3: Neural Network-Based Algorithm for Quality Detection in Boxes Stored in Workstation 2

During day-to-day work at Workstation 2, it is observed that boxes sometimes exceeded the specified height and weight. Therefore, it is proposed an intelligent system to detect those products that do not comply with the specifications.

Currently, work is performed using an AI algorithm based on neural networks to classify the product stored in Workstation 2. The product is classified as defective or non-defective according to the weight and height data of the box, obtained through the sensors attached to the band conveyor and station chassis, respectively.

**Figure 29:**
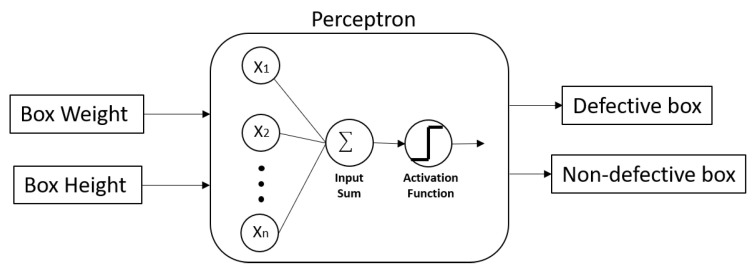
Classification model to detect defective products.

This algorithm collaborates directly to guarantee product quality (quality assurance) related to production control. Figure 29 shows the general classification algorithm model.

**Figure 30:**
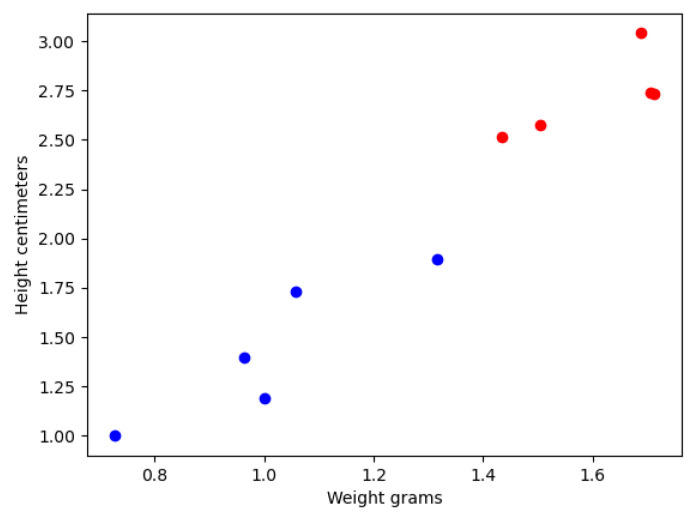
Data distribution of pieces with good quality and bad quality, according to their weight and height.

The mathematical representation of the Perceptron is:

Y = Wx + b(1)

where Y represents the output of the classification model, W represents the set of coefficients or weights of the model, and b is the bias to trigger the Perceptron’s output. The use of a single perceptron model was chosen due to the data distribution that, in this case, is linearly separable, as shown in Figure 30. The blue points represent a non-defective piece, and the red ones are defective production pieces. This data was taken from Workstation number 2 through the SCADA system.

For training the Perceptron model, it was set the number of epochs to 1000 and the learning rate to 1. The above data was divided into three segments: training, validation, and data testing, to avoid overfitting. The obtained perceptron model generated from Figure 30 data can be seen next (Equation (2)):

Y = [0.90958486, −0.816145]x − 1(2)

If the output value Y is equal to or greater than 0, the new piece x is classified as a defective piece; conversely, if the output value of y is less than 0, the value is classified as a non-defective piece. The activation function chosen for this purpose was a single unit step. Due to the simplicity of the model and the data, it could be embedded this model into a microcontroller with low computational requirements, thereby enhancing the inspection process of the pieces for the operator and improving the system’s manufacturing process.

## 6. Conclusions

This paper proposes a reference manufacturing AP model based on the DT concept. The main objective of proposing a new model is the continuous evolution of manufacturing systems incorporating new structures and smart technologies for more flexibility in enterprise operations. Thus, the entire manufacturing system could increment its robustness. Besides, a fast response between the pyramid levels could be achieved.

Emerging technological structures such as DT, manufacturing systems can evolve into dynamic systems that offer assistive solutions for decision-making at strategic points in the production chain. In addition, through incorporating artificial intelligence algorithms, new manufacturing systems can autonomously improve their performance securely and supervised.

Regarding the case study, undergraduate and graduate students can access learning resources that they do not have available offline by applying new technologies. Those technologies can be run online. Although each application corresponds to a DT of the proposed model, and despite the larger-scale scope that the DT-based AP model may have, the work presented permeates the relevance of the proposed model and the importance of filling new technological gaps.

For future work, it is essential to complement each application in the case study to achieve the proposed model in its entirety. In this way, the proposed theoretical model can be reliably evaluated. Sporadically, the development of DT-3 should be thoroughly worked on, which will complement the rest of the work carried out, corresponding to DT-1 and DT-2. In this way, users can have a complete manufacturing system that can be a radically valuable tool for training with a highly technological, avant-garde manufacturing system.

## Figures and Tables

**Table 1:** Production Scheduling (PS) Details.

Production Scheduling (PS)	
Functions	The information generated or modified by (PS)
**Determine the production schedule.**	The production schedules.
**Identify long-term raw materials requirements.**	The actual production versus the plannedproduction.
**Determine pack-out schedule for end products.**	The production capacity and resource availability.
**Determine available products for sales.**	Current order status.

**Table 2:** Variables in OPC server and LabVIEW.

Name in OPC Server	Name in LabVIEW Environment	PLC Direction	Description
**Q1_1**	Extended Piston	Q1.1	Through this variable, it is possible to monitor the piston position
**M23_0**	Enter Button	M23.0	Through this variable, the production is started
**I1_1**	Box sensor	I1.1	Through this variable, it is possible to monitor the sensor state, which detects the boxes

## Data Availability

Not applicable.
